# Emotional Intelligence: An Untapped Resource for Alcohol and Other Drug Related Prevention among Adolescents and Adults

**DOI:** 10.1155/2012/281019

**Published:** 2012-04-18

**Authors:** Ken Russell Coelho

**Affiliations:** Department of Psychology, University of California, Berkeley, CA 94720, USA

## Abstract

Alcohol and Other Drug abuse in adolescents and adults continues to be a major public health problem in the United States. Care in intervention programs aimed at high risk populations identified occurs after the maladaptive behavioral delinquency has occurred, and only then is an individual afforded the opportunity to join an intervention program. The focus of this paper is to illustrate and highlight the value of prevention programs which emphasize altering maladaptive behavior before the behavior becomes problematic. Emotional Intelligence is not only an indicator of alcohol and other drug abuse, but is linked to emotional competence, social and emotional learning, the development of healthy and life promoting behavior, and has been proven to reduce some of the risk factors associated with alcohol and other drug abuse in adolescents and adults. This paper seeks to recognize the significance of Emotional Intelligence as a desirable health promoting attribute and to establish the importance of its conceptual use in a prevention based model for reducing associated high risk behaviors.

## 1. High Risk Behavior in Adolescents and Adults

Alejandro is a 12 year old adolescent who lives in the inner city of West Oakland in Northern California. He lives in an area rampant with crime, drugs, violence and prostitution. His immigrant parents were offered asylum refugee immigration status to permanently reside in the United States twelve years ago. Alejandro is the first and only child in his family to be born in the United States. He attends high school where he gets into frequent trouble with authority. His parents often get phone calls from the school complaining that he cannot control himself, and gets into fist fights with other classmates. Due to these behavioral problems in school, his parents get very angry with him and get into arguments with each other quite often, which usually leads to Alejandro's father turning violent and either destroying furniture or physically abusing Alejandro. This might be due in-part to the fact that Alejandro's father is an alcoholic and has a pack a day history of smoking. His parents are not used to open communication with him, instead they just address issues by declaring that he should simply stop indulging in disruptive behavior in school, without asking him as to why and what made him resort to these delinquencies. They compare him to other well-behaved children in the community to make him feel guilty about his acts of violence which causes him to alienate and distance himself from them. As a result, Alejandro does not confide in his parents and avoid them, the result being even more violence, frequent fights with other students, disrespect to teachers and class disruptions. He is now a member of the drug ring at school that spends time after school smoking marijuana. On some days, Alejandro steals a bottle of his father's alcohol for his friends to drink. Alejandro is now much more violent in the park with other peers who indulge in and encourage these maladaptive behaviors together. Due to these activities, his academic performance is deteriorating and is on probation. Initially, the problem was behavioral, but due to the lack of effective communication, understanding and support from his family and school environment, it has now escalated to drug and alcohol abuse, and it is affecting his life.

This is a classic example of how generalized patterns of delinquent or abusive behaviors which, when unaddressed can escalate into drug or alcohol related problems. Unfortunately, it is only when an individual indulges in the addiction or abuse, that the behavior is determined to be high risk and afforded the opportunity to be a part of an intervention program that effectively treats the alcohol and other drug (AOD) abuse and addiction. Efforts to curb high rates of AOD abuse should focus on prevention programs which emphasize altering maladaptive behavior before becoming problematic. This is extremely relevant to the case presented. If Alejandro was given the right tools to effectively confront these behavioral challenges with his family and school, he would be had been equipped to handle these problems before they escalated to the point of drug abuse. Developing an effective prevention program will prove useful in creating a cadre of highly aware and competent adolescents empowered with relevant knowledge and the right guidance to make informed decisions.

The institution of AOD prevention training and intervention programs for adolescents should focus on the education and implementation of emotional intelligence (EI) as a core competence for adolescents in high schools. The key is to provide our adolescent community an umbrella skill set that would afford the opportunity to better serve their growing and developing needs thereby empowering themselves to develop better personal family and peer relationships which would in-turn help adolescents ward off indulgence in health damaging and risky behaviors and encourage healthy lifestyles. EI would help create the foundation for prevention programs of the future, focusing on emotional regulation and maintenance strategies for the attainment of positive health outcomes. Goleman, in his much acclaimed article titled “What makes you a leader?” published in the Harvard Review Best of 1998, explains that much of life's successes and ability to ward of potential harm to oneself can be achieved by increasing ones self awareness, self regulation, motivation, empathy, and social skills. Developing these five dimensions of EI impove individual's accuracy at recognizing and understanding one's emotions, affording the opportunity to manage and regulate the emotional expressions of themselves and those of their peers, family members and people around them. The ability to recognize, perceive, and regulate emotional expressions will in turn motivate them to be more aware of themselves, their behavior and those around them, regulate their own behaviors, moods and those of others and encourage them to behave in more conducive manner, thereby reducing the potential risk of developing AOD problems in the future.

Core focus of this paper is to bridge together the current literature on EI, social emotional learning (SEL), emotional competence and AOD abuse in adolescents and adults in order to recognize the significance of EI as a desirable health promoting attribute and to establish the importance of its conceptual use in a prevention based model for reducing high risk behaviors associated with adolescent and adult AOD abuse.

## 2. Review of Emotional Intelligence

EI has been described many times in psychological theory. EI was first formerly introduced to the scientific body of literature through the cutting edge work of Salovey and Mayer in 1990 and was initially defined as the ability to understand feelings within oneself and in others and to use these feelings in guiding thought and action [1]. Any thought on EI before the 1990s was not in the domain of psychology but was introduced and applied to other domains such as anthropology, evolutionary sciences, cognitive affective study areas, and the like.

Although Salovey and Mayer's discussion of EI was the first and as a result of which, led to a lot of excitement and discussion in the field, leading to many definitions of EI, one of which is significant to our discussion because it pushed the research community to identify EI as a multi-dimensional construct while, thoroughly hallmarked potential applications to other fields; for example, Daniel Goleman's book published in 1995 titled “*Emotional Intelligence: Why it can matter more than IQ?*” Because of EI's surging applications to a variety of settings, it has become increasingly important as a predictor of life success and workplace productivity that it accounts for much of human success than other traditional predictors of success such as IQ [2]. He contended that EI explained up to 80% of unaccounted successes in life and that the remaining was mainly due to technical competence accounted by differences in IQ. Goleman defined EI as “the abilities to motivate oneself, persist in the face of frustrations, regulate ones moods, emotions and behaviors, empathize with other people and to be able to maintain an above average level of social skills” [2]. In this paper and for the purpose of discussion, EI is defined and identified by Goleman because firstly, his overarching and inclusive definition of EI permits its use as a multi-dimensional construct allowing for application to other fields and in health intervention and prevention programs which would benefit from the ability to perceive and regulate emotional expressions and secondly, Goleman's publications continue to be the definitive reference on the subject [3, 4]. We will also briefly discuss the relationship between EI, social emotional learning (SEL) as well as the relationship between emotional competence, substance abuse and high risk behavior.

## 3. Emotional Intelligence and Social Emotional Learning

Central to the concept of emotional intelligence is the role of social and emotional learning which integrates competence promotion and youth development frameworks for reducing risk factors and assists in fostering protective mechanisms for positive adjustment [5–8]. Researchers in the field have defined SEL in various ways; however, Waters and Sroufe's initial explanation in 1983 described competent people as those who have the abilities “to generate and coordinate flexible, adaptive responses to demands and to generate and capitalize on opportunities in the environment.” SEL was later defined as the process of acquiring core competencies to recognize and manage emotions, set and achieve positive goals, appreciate the perspectives of others, establish and maintain positive relationships, make responsible decisions, and handle interpersonal situations constructively [9]. SEL programs focus on the development of five interrelated sets of cognitive, affective, and behavioral competencies to include self-awareness, self-management, social awareness, relationship skills, and responsible decision making (CASEL: Collaborative for Academic Social and Emotional Learning, 2005). These competencies in turn provide a foundation for an improved adjustment and academic performance as reflected in more positive social behaviors, fewer conduct problems, less emotional distress, and improved test scores and grades [10]. Over time, mastering SEL competencies result in a developmental progression that leads to a shift from being predominantly controlled by external factors to acting increasingly in accord with internalized beliefs and values, caring and concern for others, making good decisions, and taking responsibility for one's choices and behavior [11]. The collaborative for academic social and emotional learning marked the first attempt in using the words *social and emotional learning* to describe the systematic teaching and learning of social and emotional competencies in applied academic settings. However, both the definitions of EI and SEL have commonalities, that is, both include coordination of cognition, affect, and behavior including the awareness and management of one's own emotions and awareness and understanding of others' emotions [12].

## 4. Emotional Competence, Substance Abuse and High-Risk Behavior

Living a healthy life can mean very different things for every individual. When we think of health behaviors, what does living a healthy life really mean? Definitions for a healthy life should consider the impact of one's own actions and its effect on oneself and as well as of those around us. These judgments and behaviors affect the social relationships we have both with ourselves as well as with those around us [13]. When a friend chews tobacco or even smokes around us, it affects the interaction together, or when a classmate in school decides to skip class and head over to the local pub, it affects the peer-to-peer interactions in school for the day and introduces the element of delinquencies in behavior, also directly influencing the relationship with family and teachers. In this manner, poor health choices and poor social and emotional competence clearly affect the individual and interactions with their friends, family, and social circle.

There is an ongoing recognition which has increased over the recent years, of the importance of individual skill building in the areas of decision making, problem solving and communication as part of school health-based prevention programs aimed at reducing high-risk health behaviors. Dalton et al. [14] in his work on *linking individuals with communities* recognizes the ecological impact of these high-risk behaviors. Daniel Goleman in his book on emotional intelligence also reveals ways in which high-risk behavior prevention may prove useful to reduce the high rates [2]. The value of such individual' EI skill-building as prevention tools has seen itself best utilized in the field of education, whereby teachers and educators have the potential to build a cadre of knowledgeable, responsible, caring, nonviolent, healthy individuals with thoughtful sustained and systematic attention to the social and emotional life of children and youth [9, 15]. 

Emotional competence and social emotional learning both reinforce and support efforts to prevent alcohol and other drug use, violence, and other problem behaviors that traditionally serve as barriers to learning as well as conduits to the promotion of high-risk behaviors. According to Elias et al., 1997 in *Promoting Social and Emotional Learning: Guidelines for Educators,* the development of both these social and emotional attributes is essential for building and sustaining life-long relationships which are important for leading a healthy life, academic success, a safe and civilized classroom, and key to creating inclusive public communities.

## 5. Linking Emotional Intelligence to Alcohol and Other Drugs

Developing EI skills are advantageous in personal life and numerous occupational fields such as education, business, politics, and healthcare. EI has been identified to be of significant importance in AOD problems with adolescents and adults. The following constitute the empirical evidence linking EI to AOD.

Firstly, the lack of EI has been identified and documented as a potential indicator of AOD abuse in adolescents and adults. A study conducted at the University of New England in Australia revealed that a person's score on a test of EI predicted their potential problems with alcohol and other drugs [17].. Lower EI scores were correlated with poor coping strategies, and a person's poor coping strategies in turn predicted one's ability to involve themselves in AOD problems. However, coping strategies contributed very minutely as a mediator variable, while other main attributes of EI such as motivation, self regulation, self awareness and social skills were major mediators for the predicted outcome of AOD problems. As a result, ones ability to accurately express, perceive, recognize and regulate emotional expressions was a predictor of whether the person had alcohol and other substanceor drug abuse.

Secondly, EI has been linked to the development of healthy and life promoting behaviors. A longitudinal study conducted on a large diverse group of 6th graders in Los Angeles, California demonstrated that EI has been shown to reduce an adolescent's intentions of smoking and ultimately inhibit the behavior that causes the act of smoking [3]. The study also revealed that adolescents, who had a high degree of hostility or aggressiveness, were more likely to have lower EI, and were more likely to smoke. Also, adolescents who had a lower level of ability to refuse a cigarette from someone they had just met, was correlated with low EI. Hence, EI acts as an indicator of potential drug use and having a high degree of EI is associated with healthy life promoting behaviors which make it a great skill to adopt. Also, EI has been proven to enlighten and motivate change in delinquent AOD behaviors of a group of adolescents. A community intervention program conducted by a research group in Miami, Florida examined the effect of AOD in juvenile offenders and the study demonstrated that a motivational cognitive behavioral intervention program which focused on all of the EI attributes discussed here, namely, self-awareness, self-regulation, motivation, empathy, and social skills, guided self-change in these adolescents that were being treated for AOD problems, providing yet another compelling reason for its health promotion effects [18].

Thirdly, EI has been linked to the reduction in the risk factors that have been traditionally associated with AOD in adolescents and adults. One major risk factor that has been associated with the development of alcohol use, abuse, and continuity of use contributing to relapse of the condition of alcoholism is stress [19]. An increase in stress is a major contributor to the onset of AOD use in adolescents and adults. According to a study conducted by the psychology department of Nottingham Trent University in the United Kingdom, a high degree of EI was associated with lower stress levels and fewer psychological symptoms pertaining to traumatic experiences [20]. Another study revealed that students scoring high on a test of EI were better equipped to deal with and recover from stress and hence were less likely to fall sick or be vulnerable to the conditions brought upon by stress [21]. As a result, developing EI skills serves as a protective force, lowering one's susceptible risk to the factors that have traditionally predicted AOD use and abuse.

These reasons explain the implications of EI skills in adolescent and adult AOD abuse. The implications of imbibing EI skills in both adolescents and adults are numerous and would serve to enhance health promotion skills, reduce the risk factors that have traditionally been associated with high risk AOD use and would provide an accurate means of measuring one's inherent vulnerability to AOD problems by accessing their EI scores through a EI skill test.

## 6. Incorporating Emotional Intelligence into Alcohol and Other Drug Prevention

According to Goleman, EI's multidimensional construct is a contribution of five dimensions; self-awareness, self-regulation, motivation, empathy and social skill [16]. A summary of definitions and applications to adolescent and adult AOD prevention are provided in Table 1.

The dimension of self-awareness provides an individual with the ability to recognize and understand one's own moods, emotions, and drives as well as their effect on others. An individual high in self-awareness is more likely to be attuned to their family values, to be able to speak clearly, accurately and more objective about their emotions and the impact they have on academic performance, family life and social support which ultimately assist in dealing with AOD problems. Research studies demonstrate that low bonding, conflict with family and academic failure contribute to the individual and interpersonal factors predicting early onset of drug use [22]. Self-aware adolescents and adults are comfortable talking about their limitations and strengths in these regards and exude a high degree of self confidence when making decisions about family issues, peer pressure and drug use since they are highly aware of the condition and capabilities for improvement and are less likely to set themselves up for failure.

The dimension of self-regulation provides the person with the ability to control or redirect disruptive impulses and moods or the propensity to suspend judgment in order to think before acting. Adolescents and adults high in self regulation are better able to create an environment of trust, integrity, and fairness with themselves, the people around them, their family, and newly found social support groups that help regulate AOD abuse thereby reduces miscommunication and increases productivity in the relationship.

Association with drug-using peers, poor impulse control and early and persistent problematic behaviors have been shown to predict AOD abuse in adolescents and adults [22]. Hence, those exuding a high sense of trust and respect know when to step away when in an argument with a friend, family member, and drug or alcohol user. Also, self regulation also provides the adolescent and other adults with constructive skills to dynamically change approaches to better serve their growing personal needs and to learn to be open to making changes in life with limited drug use, or methadone maintenance treatment.

The dimension of motivation provides the person with a passion to work for more than materialistic reasons and to work for the propensity to pursue goals with energy and persistence. Adolescents and adults high in this kind of motivation exude an increased level of energy and optimism, passion for their work by seeking out frequent challenges, being a constant lifelong learner, and taking great pride in a high-level performance. Motivation provides the best environment for adolescents and other adults to reduce or cease use of drugs or disclose problems to family and support groups even in the face of numerous challenges. Alcohol and drug behavior problems and attitudes existing within the family have been shown to correlate with high potential for drug abuse in adolescents and adults [22]. Motivated adolescent and adults are more likely to be optimistic even when there are low hopes of success and are more likely to go beyond the call of duty even if it means trying to convince their peers and/or family members to cease AOD use and abuse leading to the potential cessation of the use of AOD.

The dimension of empathy provides the adolescent and adult with the ability to understand the emotional makeup of others and provides the skills in treating others around them according to their emotional reaction. A person with a high degree of empathy use the information about their families, friends, or social support group's feelings in order to be more intuitively receptive to the way they feel. They are more likely to be able to identify with others better, understand what they are going through, and be more sensitive to friends or family members who are emotionally & physically affected by AOD abuse. Since individuals with a high level of empathy are more likely to be compassionate when dealing with others in their life that might provoke them to be angry or aggressive.

The dimension of social skills provide the individual with the ability to manage effective relationship patterns and behaviors and build networks with the ability to find common ground and build rapport with family, friends and peer groups. This skill also provides a means to be effective in forming bonds with family members and close friends who do not use AOD. Also, the formation of effective family bonds and therapeutic peer support groups have been documented as protective factors for AOD problems in adolescents and adults later in life [22]. Hence, this dimensional attribute is an outcome of all the dimensions of EI and has a direct influence on use of EI in AOD related problems in adolescents and adults, thereby influencing overall health outcomes.

Each of these dimensions give meaning to EI and provide the foundational groundwork in the process of defining EI. More importantly, the five dimensions afford us the means to objectively use EI as a measure of adolescent and adult ability to actively cope and deal with AOD abuse and to be able to use this information to improve health outcomes.

## 7. Emotional Intelligence as a Desirable Health Promoting Attribute

It is clear that EI is a significant and desirable health promoting attribute and its conceptual use in a prevention based model for reducing high risk behaviors associated with adolescent and adult AOD abuse is the need of the hour. Even though the implications of EI in prevention efforts for adolescent and adult AOD abuse is clear, it is important to understand and acknowledge the need for more work in order to create effective prevention programs which focus on identifying maladaptive behaviors which encourage AOD abuse and curb these behaviors as early as possible. Firstly, developing more accurate means of measuring EI, emotional competence and SEL in adolescents and adults is necessary in order to be able to proactively identify risk groups for potential AOD use problems and prospective abuse potential. The current use of self-report and EI questionnaires as a measure of EI in a quotient (EQ) is highly inaccurate and prone to assess skewed information. Such measures are prone to obtaining false information since people's ability to self disclose is always protected by ones right to privacy and or information from third parties such as parents and co-workers also prove to be extremely meaningless, because they tend to prevent sharing or underreport a lot of information about their child or co-worker [23]. Secondly, accurate and objective EI test measures need to become a part of the mainstream middle and high school testing procedures and also at the adult workplace in order to assess for high risk potential of AOD use and abuse. Lastly, risk-specific group based EI prevention programs should be implemented in school and workplace environments with a focus on the five dimensions, of the multidimensional construct in the hope of ultimately discouraging health compromising behaviors, preventing prospective AOD abuse in adolescents and adults.

## Figures and Tables

**Table 1:** Emotional intelligence applications to AOD prevention.

Dimensional component of emotional intelligence	Definitions [16]	Examples of applications
Self-awareness	The ability to recognize and understand one's own moods, emotions, and drives as well as their effect on others.	(1) Confidently making decisions about family issues, peer pressure and drug use. (2) Awareness of family values (3) Recognizing the effect of frequent drug use on family life, academic performance and social support.

Self-regulation	The ability to control or redirect disruptive impulses and moods or the propensity to suspend judgment in order to think before acting.	(1) Knowing when to step away during an argument with a friend, family member, and drug or alcohol user. (2) Learning to be open to making changes in life with limited drug use or methadone drug replacement or maintenance treatment. (3) Developing a sense of trust and integrity with oneself, family and newly found social support group that help deal with regulating drug use.

Motivation	A passion to work for reasons that go beyond money or status or a propensity to pursue goals with energy and persistence.	(1) Providing the best environment to reduce or cease use of drugs or disclose problems to family and support groups even in the face of numerous challenges. (2) Going beyond the call of duty even if it means trying to convince peers to cease AOD use. (3) Being optimistic even when there are low hopes of success.

Empathy	The ability to understand the emotional makeup of other people or the skills in treating people according to their emotional reactions.	(1) Being understanding and inclusive in thinking of the family's perspective when making decisions. (2) Being compassionate when dealing with other people in your life that might provoke you to be angry or aggressive. (3) Being sensitive to other friends or family members who are emotionally and physically affected by your AOD use.

Social-skill	Proficiency in managing relationships and building networks or the ability to find common ground and build rapport.	(1) Being effective in forming bonds with family members and close friends who do not use AOD. (2) Being an effective member of a social support group and friends that help focus on curbing AOD abuse.

## Abbreviations

EI: Emotional intelligence
EQ: Emotional Quotient
SEL: Social and emotional learning
AOD: Alcohol and other drugs.
CASEL: Collaborative for Academic Social and Emotional Learning
